# Ethnobotanical study of traditional antivenom treatments in Burkina Faso

**DOI:** 10.1186/s41182-025-00773-x

**Published:** 2025-07-22

**Authors:** Moumouni Bandé, Abdoul Karim Sakira, Noé Rigobert Zangré, Hyacinthe Wendégoudi Bonkoungou, Evance Brice Zoungrana, Cédric Delporte, Pierre Van Antwerpen, Touridomon Issa Somé

**Affiliations:** 1 Université Joseph KI-ZERBO, Ecole Doctorale Sciences de la Santé, Laboratoire de Toxicologie, Environnement, Santé, Ouagadougou, Burkina Faso; 2 ULB - Faculty of PHARMACY, RD3 - Pharmacognosy, Bioanalysis & Drug Discovery Unit & Analytical Platform of the Faculty of Pharmacy, Bld Triomphe, Campus Plaine, CP 205/5 B-1050 Brussels, Belgium; 3 Ministère de la Santé, Direction Générale de l’offre des Soins (DGOS), Direction de la Médecine Traditionnelle et Alternative, Ouagadougou, Burkina Faso

**Keywords:** Ethnobotany, Snake, Envenomation, Plants, Burkina Faso

## Abstract

**Background:**

Snakebite envenomation constitutes a major public health challenge in Burkina Faso, particularly within rural communities. Limited access to formal healthcare services, coupled with the high cost of antivenom treatment, has led to widespread reliance on traditional health practitioners (THPs). This study was therefore undertaken to generate empirical data on the role of THPs in the management of snakebite envenomation, with a focus on the medicinal plants employed, methods of remedy preparation, and routes of administration.

**Method:**

A preliminary survey was conducted to identify traditional health practitioners THPs involved in snakebite envenomation management within the study regions. The preliminary survey involved 799 individuals selected through convenience sampling in local markets. Ethnobotanical data were subsequently gathered from the identified practitioners via semi-structured interviews. The collected data were entered and analysed via an Excel spreadsheet. In addition to the sociodemographic characteristics of THPs, the relative frequency of citation (RFC) were also determined.

**Results:**

The results revealed that 90% of the THPs were male and that 76.67% illiterate. Over half of the THPs had more than two decades of experience. Diagnosis was mainly based on symptoms, with 60% relying on bite site examination. Most treatments involved plant-based powders (73.33%), typically applied subcutaneously through incisions. Roots were the most commonly used plant part in antivenom preparations (29%), while dried and calcined materials were the predominant form of medicinal plant preparation in the region. The study identified 29 plant species across 18 botanical families. *Annona senegalensis Pers., Nauclea latifolia Sm., and Vitellaria paradoxa C.F. Gaertn* had the highest relative frequency of citation (RFC), each at 10%. Encouragingly, 63.33% of THPs had participated in training or awareness sessions with health centres. Additionally, 46.66% referred patients to hospitals when traditional treatments were insufficient.

**Conclusion:**

These results highlight the therapeutic potential of local medicinal plants in the treatment of snakebite envenomation and support the need for strengthened collaboration between traditional and biomedical healthcare systems.

## Background

Burkina Faso, a landlocked country in West Africa, is characterised by a hot, arid climate and dense tropical vegetation, particularly in its eastern and southern zones [1]. This ecological setting supports a high diversity of venomous snake species, with members of the Viperidae family being the most prevalent [2]. The population is predominantly rural and youthful, with widespread engagement in farming and livestock rearing. These activities frequently bring individuals into contact with grasslands, forested areas, and snake habitats, thereby contributing to a high annual incidence of snakebite cases. Between 2010 and 2020, an estimated 213,683 snakebite cases were recorded [3]. This corresponds to an annual incidence exceeding 20,000 cases, with a case fatality rate of approximately 3% [4].

Snakebite envenomation is recognized as a serious medical emergency [5]. It typically presents with local symptoms such as erythema, oedema, and intense pain at the bite site, usually within the first hour. Systemic manifestations may involve emetic episodes, visual disturbances, paraesthesia of the extremities, and diaphoresis [6]. The limbs and hands are the most commonly affected areas. Psychological reactions commonly observed among envenomated patients include acute anxiety, which is often associated with tachycardia and presyncope [5]. The severity and specificity of clinical manifestations depend largely on the species of snake involved, the quantity of venom injected, and the individual physiological response to envenomation [7].

The treatment of snakebite envenomation typically involves symptomatic management using anti-inflammatory, analgesic, and anticoagulant and sedative therapies [8]. The administration of antivenom serum significantly improves survival outcomes [9]. However, antivenom production is complex and resource-intensive. It requires the preparation of venom mixtures for immunization, the generation of hyperimmune plasma, and the purification of immunoglobulins [10]. In many low-resource settings, fragile infrastructure and high production costs, limit the availability and accessibility of antivenom [11]. As a result, many affected individuals, particularly in rural areas, rely on traditional herbal remedies [12]. These treatments are more accessible, can be used against multiple snake species, and do not require cold storage [13].

Scientific investigations have identified a range of phytochemicals exhibiting antivenom activity. Plant-derived extracts have thereby shown efficacy in counteracting the inflammatory, haemorrhagic, myotoxic, and neurotoxic effects induced by snake venom. In certain cases, these extracts are also employed prophylactically to mitigate the risk of complications [14–17].

In light of the growing interest in antivenom phytotherapy, this study was undertaken to document ancestral knowledge that is traditionally transmitted orally and increasingly at risk of being lost. Specifically, it generates scientific data on traditional health practitioners involved in the management of snakebite envenomation, investigates the medicinal plants they use, the methods of remedy preparation, and the routes of administration.

## Materials and methods

### Description of the study area

**Fig. 1:**
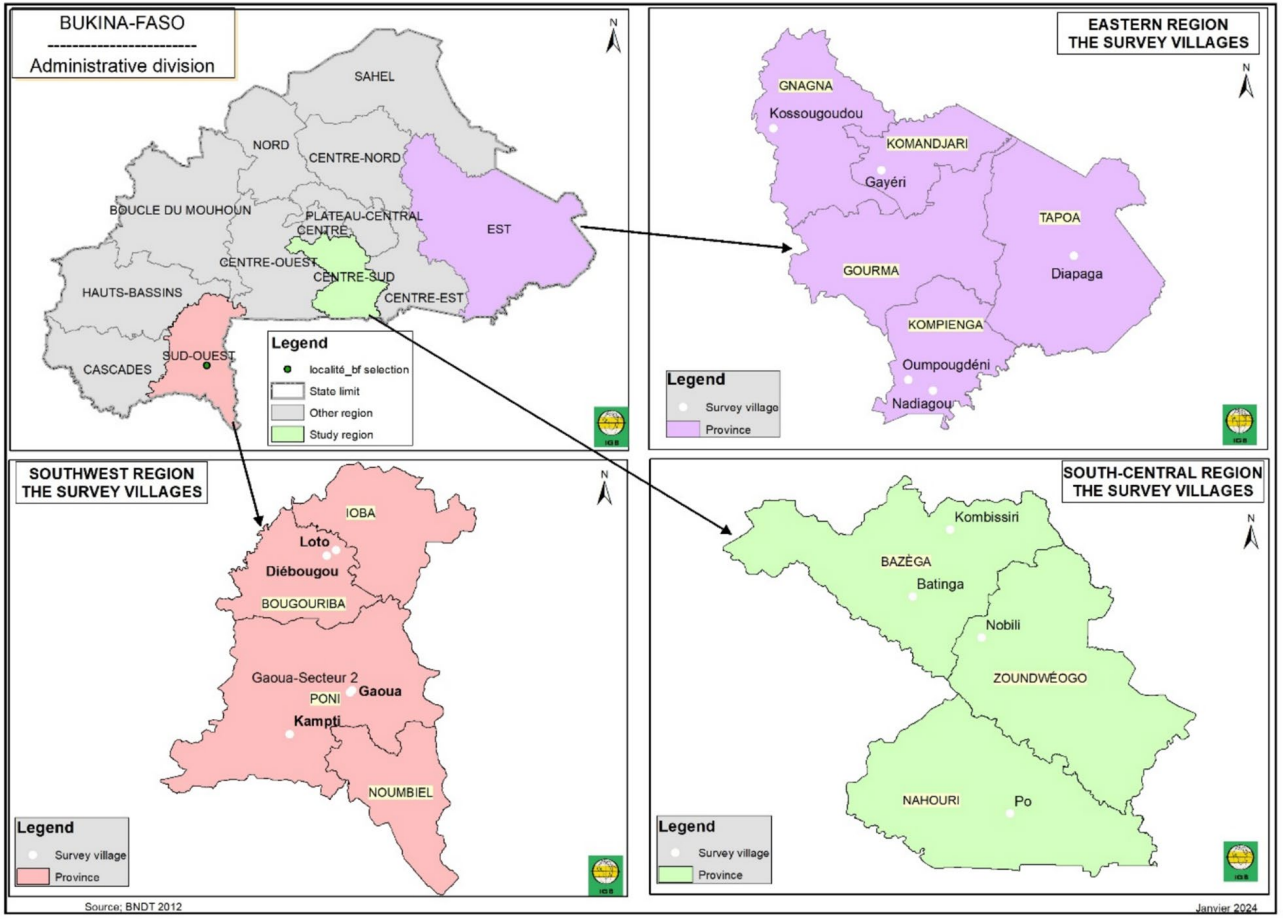
Geographical location of study regions

This research constitutes a prospective ethnobotanical study conducted within the Eastern, Centre-Southern, and South-Western health regions of Burkina Faso (Fig. 1).

The Eastern Region is the largest administrative area in Burkina Faso, covering 46,694 km^2^. The Centre-South region occupies 11,457 km^2^, whereas the South‒West region spans 16,318 km^2^. These regions comprise five, two, and three provinces, respectively [18].

In terms of population, the Eastern region had an estimated 1,941,505 inhabitants in 2019. The Centre-South region recorded 788,341 inhabitants, and the South‒West region was home to approximately 874,030 people during the same year [19].

In the Eastern Region, fieldwork was conducted in the localities of Nadiagou, Oumpougdeni, Diapaga, Gayéri, and Kossougoudou. In the Centre-South Region, data collection took place in Kombissiri, Batinga, Nobili, and Pô. In the South-West Region, surveys were carried out in Loto, Diébougou, Gaoua, and Kampti (Fig. 1). These three regions were purposively selected to capture potential inter-regional variations in traditional practices related to the management of snakebite envenomation. Moreover, the Eastern, Centre-Southern, and South-Western health regions report thousands of snakebite incidents annually. Between 2010 and 2020, a total of 35,214 cases of snakebite envenomation were recorded by health services in these regions [3], representing an average of over 3000 cases per year.

### Informant selection and sampling methods

A preliminary survey was conducted to identify traditional health practitioners involved in snakebite envenomation management within the study regions. Interview teams, each composed of four members, were deployed to the central markets of the main towns in the three selected regions. These included the markets of Fada (Eastern Region), Manga (Centre-South Region), and Gaoua (South-Western Region).

A convenience sampling method was used. Each interviewer followed a single direction through the market and approached consenting adults for participation. A total of 799 individuals were interviewed: 249 in the East, 283 in the Centre-South, and 267 in the South-West.

The questionnaire included the following key questions:

- Are you aware of a traditional health practitioner who treats snakebite envenomation?
- Have you or someone you know consulted such a practitioner?
- Do you know anyone who recovered from snakebite envenomation using only traditional remedies?

If the first two questions were answered affirmatively, further details were collected. These included the practitioner’s full name, location (village or town, sector or district), and telephone number, if available.

Following the survey, the ten most frequently cited practitioners were selected for the ethnopharmacological investigation. The research team visited each practitioner in their respective village to conduct in-depth interviews and field observations.

### Data collection from traditional health practitioners

Data were collected using a standardised survey protocol based on a semi-structured questionnaire. The questionnaire included both open- and closed-ended questions. It was initially drafted in French, then translated into the local languages: Goulmatchema, Mooré, and Dagara, for the Eastern, Centre-Southern, and South-Western regions, respectively. Local interpreters assisted with translation when needed.

Traditional health practitioners were interviewed about their knowledge and practices concerning the use of medicinal plants for treating snakebite envenomation. The questionnaire was divided into two sections. The first section gathered sociodemographic information. The second focused on ethnobotanical data, including vernacular plant names, plant parts used, preparation and administration methods, and the sources and transmission of knowledge.

Plants cited by practitioners under local names were identified in situ by a botanist. The scientific names of all recorded species were verified using the World Flora Online database (http://www.worldfloraonline.org, accessed on 27 May 2025).

### Data analysis

The data collected in collaboration with the Department of Traditional Medicine, Pharmacopoeia, and Alternative Medicines of the Ministry of Health of Burkina Faso were entered into a microcomputer via a Microsoft Excel (2010) spreadsheet, which also served as the primary tool for data analysis.

On the basis of the recorded data the relative frequency of citations (RFC) for each plant species were calculated by dividing the number of informants who mentioned a particular species (FC) by the total number of informants surveyed (N) [20]: RFC = FC/N.

### Ethical considerations

Prior to the interviews, the objectives of the study were clearly communicated to the participants, who subsequently provided their informed consent. To ensure confidentiality, all survey forms were anonymised.

## Results

### Traditional health practitioners selection

**Fig. 2:**
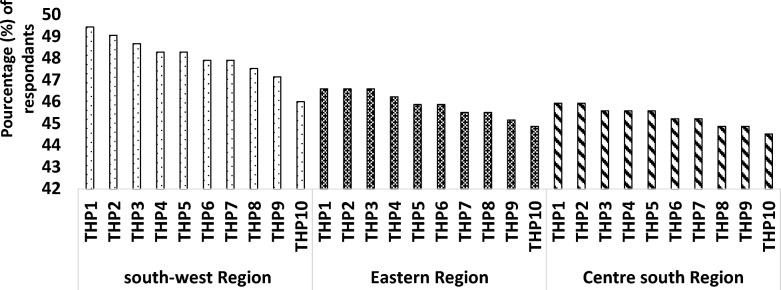
Distribution of respondents by traditional health practitioners (THPs) and region

The percentage distribution of the respondents is presented in Fig. 2.

The findings showed that, in each of the three surveyed regions, at least 40% of the respondents recognised the ten most reputed traditional health practitioners (THPs) for treating snakebite envenomation. In the southwestern region in particular, this recognition ranged from 46% for the least-cited THP to 49.4% for the most frequently mentioned THP.

### Perceptions of the efficacy of traditional health remedies

Table 1 presents the distribution of survey participants from each of the three regions, categorised according to their views on the traditional management of ophidian envenomation and the perceived effectiveness of the remedies.

**Table 1:** Respondents’ perceptions of the effectiveness of antivenom recipes provided by traditional health practitioners (THPs)

Regions	Number of informants who know one of the region’s THPs	Proportion of individuals with direct or indirect experience of successful use of traditional antivenom remedies (%)
East	249	149 (59.8)
Centre-South	283	176 (62.2)
South‒West	267	165 (61.8)
Total	799	490 (61.3)

The results of the survey, showed similar proportions of informants (59.8–62.2%) that in the South-West region, 62.2% of those surveyed had direct or indirect experience of successful use of a regional THPs remedy.

### Sociodemographic profile of the THPs

The sociodemographic characteristics of the THPs surveyed in this study are summarised in Table 2.

**Table 2:** Sociodemographic data of the traditional health practitioners surveyed

Distribution of the THPs surveyed by gender		
Gender	Frequency	Percentage
Female	3	10
Male	27	90
Distribution of THPs based on their years of experience in managing snakebite envenomations		
Seniority (years)	Frequency	Percentage
[5; 10]	3	10.0
[10; 20]	10	33.3
More than 20	17	56.7
The educational level of THPs		
Primary level	7	23.3
Illiterate	23	76.7

The findings revealed that 90% of THPs surveyed were male, with more than 56% possessing over two decades of experience in the management of snakebite envenomation within their respective communities. With respect to their educational background, more than 76% of the respondents were found to be illiterate.

### Diagnosis of snakebite envenomation THPs

The various diagnostic methods employed by THPs to identify snakebite envenomation are summarised in Table 3.

**Table 3:** Diagnosis of snakebite envenomation

Diagnostic method	Frequencies	Percentages (%)
Observation of bite site characteristics (e.g., pumice-like and bloody marks, presence of fangs, scent at the bite site)	18	60.0
Analysis of clinical signs (e.g., dizziness, drowsiness, excitement, loss of consciousness, oedema, bleeding, accelerated heart rate and pulse)	8	26.7
Administration of products (e.g., to extract fangs, observation of bloody saliva postadministration, appearance of floral-shaped traces on the bitten limb)	4	13.3
Total	30	100

The most frequently employed diagnostic method by THPs was the observation of bite site characteristics, with 60% of practitioners using this approach. This was followed by the analysis of clinical signs exhibited by the victim (26.7%) and the administration of traditional products for diagnostic purposes (13.3%).

### Clinical manifestations reported by THPs

Table 4 presents the clinical manifestations reported by snakebite victims to the THPs surveyed.

**Table 4:** Frequencies of clinical manifestations of ophidian envenomation associated with THPs

Clinical signs	Citation frequencies	Percentage (%)
Signs of inflammation (pain, oedema, necrosis)	21	70
Neurological signs (dizziness, drowsiness, excitement, delirium, coma)	8	26.7
Haematological signs (haemorrhage, haemoptysis, paleness of palms and integuments)	25	83.3
Cardiovascular signs (accelerated heart rate and pulse, collapse)	12	40.0
General signs (chills, vomiting, hyperthermia, discolouration of palms and integuments)	9	30

The clinical manifestations most commonly encountered by THPs in their practice were hemotoxic symptoms, including hemorrhage, hemoptysis, and paleness of the skin and integuments (reported by 83.3% of THPs), as well as signs of inflammation such as pain and edema (reported by 70% of THPs).

### Characteristics of antivenom recipes

The characteristics related to the physical form, preparation method, administration route, proposed mechanism of action, and treatment cost of the recipes recommended by THPs for the management of snakebite envenomation are summarised in Table 5.

**Table 5:** Characteristics of the recipes used by the surveyed THPs

Physical presentation of recipes		
Features	Frequency of quotations	Percentage (%)
Liquid	2	6.7
Solid (powder)	22	73.3
Liquid and solid (powder)	6	20.0
Method of administration		
Per os (powder to suck, mixed with porridge then ingested, dissolved in water then drunk, or consumed directly for liquid recipes)	10	33.3
Subcutaneous (application to incisions made at the bite site)	20	66.7
Single-dose	18	60.0
Repeated doses	12	40.0
Claimed mechanism of action		
Neutralises toxic venom elements	10	33.3
Corrects the toxic effects of venom	6	20.0
Neutralises toxic venom elements and corrects venom’s toxic effects of venom	14	46.7
Treatment specificity		
Recipes Combatting Envenomation by a Specific Snake Species	12	40.0
Recipes Effective Against All Types of Snakebites	18	60.0
Treatment costs		
Free	4	13.3
< $10 (US)	20	66.7
≥ 10 (US)	6	20.0

The results showed that 73.3% of the remedies used by THPs were in powdered form, while 6.7% were in liquid form. The most common preparation method was calcination of plants or plant parts, reported by 66.7% of THPs.

Regarding administration, 66.7% of THPs applied the remedies locally to incisions made at the bite site. In terms of perceived mechanisms of action, 46.7% stated that their remedies both neutralised the venom and mitigated its toxic effects.

Concerning treatment specificity, 60% of THPs claimed their remedies were effective against all types of snakebite envenomation.

Finally, 66.7% reported that the cost of treatment was less than 10 US dollars.

### Collaboration between THPs and conventional healthcare services

Table 6 provides an overview of the mechanisms of collaboration between the surveyed THPs and conventional healthcare personnel in the management of snakebite victims.

**Table 6:** Distribution of THPs according to their level of collaboration with conventional healthcare staff

Level of collaboration	Frequency	Percentage (%)
No collaboration	4	13.3
Referral/Recourse to conventional therapy for patients who do not respond to traditional therapy	14	46.7
Participation in Training/Awareness-Raising Sessions Organised by Conventional Health Centres	19	63.3

The results revealed that 63.3% of the surveyed THPs had received training or awareness sessions organised by their local health districts. In terms of referral and counterreferral, 46.7% of the THPs reported referring snakebite victims to conventional healthcare centers if the patients did not respond to their treatments.

### Medicinal plant diversity

Table 7 presents the plant species employed by the THPs in the studied regions for the preparation of antivenom remedies.

**Table 7:** Regional distribution of plant species utilised in the preparation of anti-venom remedies

Regions	Plant family	Scientific name of plant species	Parts used	Preparation method
East	Asteraceae	*Ageratum conyzoides* L.	Leaves and stems	The leaves and stems are calcined, pulverised and the resulting powder is applied into incisions made around the bite site
	Anacardiaceae	*Lannea velutina* A.Rich	Roots	The roots are calcined, pulverised and three pinches (for men) or four pinches (for women) of the resulting powder are placed into some gruel, which is then given to the snakebite victim to drink
	Annonaceae	*Annona senegalensis* Pers	Leaves/roots	The leaves are Chewed and the juice swallowed, the roots are dried, carbonised, and the resulting powder is applied into incisions made around the bite site
	Combretaceae	*Guiera senegalensis* J.F.Gmel	Leaves and stems	The leaves and stems are calcined and the resulting powder is applied in the incisions around the bite site
	Fabaceae	*Senegalia macrostachya* (Rchb. ex DC.) Kyal. & Boatwr	Barks	A spoonful of bark powder is placed into tamarind juice and administered per os to the snakebite victim, while also the powder is placed to the incisions made around the snakebite site
	Meliaceae	*Entada africana* Guill. & Perr	Barks	The barks are calcined, peeled and carbonised. The resulting powder is applied into the incisions made around the snakebite site
	Poaceae	*Cymbopogon giganteus* Chiov	Leaves	The herbs are dried, carbonised, and pulverised to obtain a powder. Three pinches (for men) or four pinches (for women) are added to water and administered per os to the snakebite victim. The powder is also mixed with shea butter and apply to the bitten limb
	Polygalaceae	*Securidaca longepedunculata* Fresen	Roots	The herbs are sun-dried, pulverised and the powder is spread around the compound to act as a repellent for snakes
	Rubiaceae	*Nauclea latifolia* Sm	Leaves and roots	The leaves and roots are dried, burnt and then pulverised. A spoonful of this powder is placed into lukewarm water and administered per os to the snakebite victim. Additionally, a pinch of the powder is mixed with shea butter and applied to the bite site
	Sapotaceae	*Vitellaria paradoxa* C.F. Gaertn	Bark	The dried bark is cut into small slices. These slices are carefully burnt to obtain charcoal, then the charcoal is grinded in a mortar to obtain a fine powder. A pinch of this powder is applied to small incisions made at the site of the bite. Another pinch of the powder is placed into some water and then given to the snakebite victim to drink
South Central	Annonaceae	*Annona senegalensis* Pers	Leaves/roots	The leaves are Chewed and the resulting juice swallowed by the victim of snake bite. Alternatively, the roots are dried and pulverised. A pinch of the resulting powder is applied into incisions made on the bite site
	Apocynaceae	*Calotropis procera* (Aiton) Dryand	Roots	The roots are dried, burnt and then pulverised. A spoonful of this powder is placed into lukewarm water and administered per os to the snakebite victim
		*Saba senegalensis* (A.DC.) Pichon	Roots	The leaves are dried, calcined and then pulverised. A spoonful of this powder is placed into lukewarm water and administered per os to the snakebite victim
	Celatraceae	*Gymnosporia senegalensis* (Lam.) Loes	Roots	The roots are calcined, pulverised. Three pinches of the resulting powder are added to the slurry and administered per os to the snakebite victim
	Combretaceae	*Combretum micranthum* G. Don	Roots	The roots are sun-dried and subsequently pulverised. The resulting powder is then dispersed around the perimeter of the compound as a reptile deterrent
		*Guiera senegalensis* J.F. Gmel	Leaves	The leaves are crushed and the resulting juice is applied directly to the snakebite site
	Euphorbiaceae	*Phyllanthus virosus* (Roxb. Ex Willd.) Royle	Stems	The stems are grinded and the resulting juice is applied to incisions made in the bitten area
		*Bridelia ferruginea* Benth	Barks	The bark is calcined and pulverised. Three pinches of the resulting powder are added to some gruel and administered per os to the snakebite victim
	Liliaceae	*Allium cepa* L	Bulbs	The onion is grinded and the resulting paste is applied to the incisions made at the snakebite site
	Malvaceae	*Bombax costatum* Pellegr. & Vuillet	Barks	The bark is sun-dried, pulverised and the resulting powder is spread around the compound to deter reptiles
		*Sterculia setigera* Delile	Barks	The bark is sun-dried, then burnt sparingly to obtain charcoal. The charcoal is then pulverised and a pinch of the resulting powder is added to some gruel and given to the snakebite victim for drinking
	Poaceae	*Heteroscyphus giganteus* (Steph.) Hürl	Leaves	The herbs are dried, carbonised, and pulverised to obtain a powder. Three pinches (for men) or four pinches (for women) are added to water and administered per os to the snakebite victim. The powder is also mixed with shea butter and apply to the bitten limb
		*Eleusine indica* (L.) Gaertn	Leaves	The leaves are dried, pulverised, and three pinches are added to the slurry and given to the snakebite victim to drink
	Rubiaceae	*Gardenia ternifolia* Schumach. & Thonn	Stems	The stems are dried, burnt gently to obtain charcoal which are then pulverised. A spoonful is placed into the porridge and administered per os it to the snakebite victim
		*Gardenia erubescens* Stapf & Hutch	Leaves	The leaves are dried, calcined and then pulverised. A spoonful of this powder is placed into gruel and administered per os to the snakebite victim
		*Nauclea latifolia* Sm	Roots	The roots are carefully burned to obtain charcoal, which is then pulverised. A spoonful of the resultant powder is added to porridge and administered per os to the snakebite victim
	Rutaceae	*Zanthoxylum zanthoxyloides* (Lam.) B.Zepern. & Timler	Stems	The fresh stems are given to the victim of snakebite to chew and swallow the juice
	Sapotaceae	*Vitellaria paradoxa* C.F. Gaertn	Barks	The bark is Calcined and pulverised. Three pinches of the resulting powder are added to some gruel and administered per os to the snakebite victim. Another pinch of the powder is applied into incisions made at the bite site
Southwest	Anacardiaceae	*Mangifera indica* L	Roots	The roots are calcined, pulverised and a pinch of the resulting powder is placed into water, and given to the bite victim for drinking
	Annonaceae	*Annona senegalensis* Pers	Leaves	The leaves are chewed, the juice swallowed and the crushed leaf extract applied to the incisions made at the bite site
	Apocynaceae	*Carissa spinarum* L	Leaves	The leaves are infused with lukewarm water, and one glass of the resulting infusion should be administered to the snakebite victim three times daily for 3 days in the case of men, or 4 days in the case of women
	Ebenaceae	*Euclea divinorum* Hiern	Barks	The dried trunk are cut into small pieces, approximately 5 cm in length. These pieces are then carefully charred to produce charcoal, which is subsequently ground into a fine powder using a mortar
		*Diospyros mespiliformis* Hochst. ex. A.DC	Barks	The bark should be sun-dried, then calcined. The calcine obtained is subsequently pulverized. The resulting powder is applied to the incisions made at the bite site. Simultaneously, three pinches of the powder are added to lukewarm water and administered orally to the snakebite victim
	Euphorbiaceae	*Flueggea virosa (Roxb. ex Willd.) Royle*	Roots	The roots are calcined and subsequently pulverised. A quantity equivalent to a teaspoon is then added to millet porridge. The porridge is administered orally to the bite victim for consumption
		*Bridelia ferruginea* Benth	Stems	The fresh stems are given to the victim of snakebite to chew and swallow the juice
	Lamiaceae	*Vitex doniana* Sweet	Barks	The bark is dried, carbonised and pulverised. Three pinches of the resulting powder is added to water and administered per os to the snakebite victim
	Polygalaceae	*Securidaca longipedunculata* Fresen	Barks	The bark is sun-dried, pulverised and spread the resulting powder around the compound to deter snakes
	Rubiaceae	*Gardenia ternifolia* Schumach. & Thonn	Stems	The stems are dried, carbonised and pulverised. Three pinches are placed into millet porridge and administered per os to the snakebite victim
		*Nauclea latifolia* Sm	Roots	The dried roots are calcined and pulverised. Three pinches of resulting powder are added into and the mixture is administered per os to the snakebite victim
	Sapotaceae	*Vitellaria paradoxa* C.F.Gaertn	Trunk	The trunk is sun-dried, carbonised and then pulverised. The resulting powder is applied into incisions made at the site of the bite. An equivalent of a tablespoon is placed into water and given to the snakebite victim for drinking

A total of 29 distinct plant species from 18 different plant families were used by traditional health practitioners to prepare traditional antivenom recipes. Three plant families, i.e., Annonaceae, Rubiaceae*,* and Sapotaceae, were common across all three study regions. The plant family most frequently involved in the preparation of antivenom recipes was Rubiaceae, with five plant species, followed by Euphorbiaceae, with four species. Annonaceae, Apocynaceae, Poaceae, and Sapotaceae each contributed three plant species. The plant species *Annona senegalensis* Pers*., Nauclea latifolia* Sm., and *Vitellaria paradoxa* C.F. Gaertn were used in all three regions to prepare antivenom recipes.

### Plant part used and initial processing of plant material

**Fig. 3:**
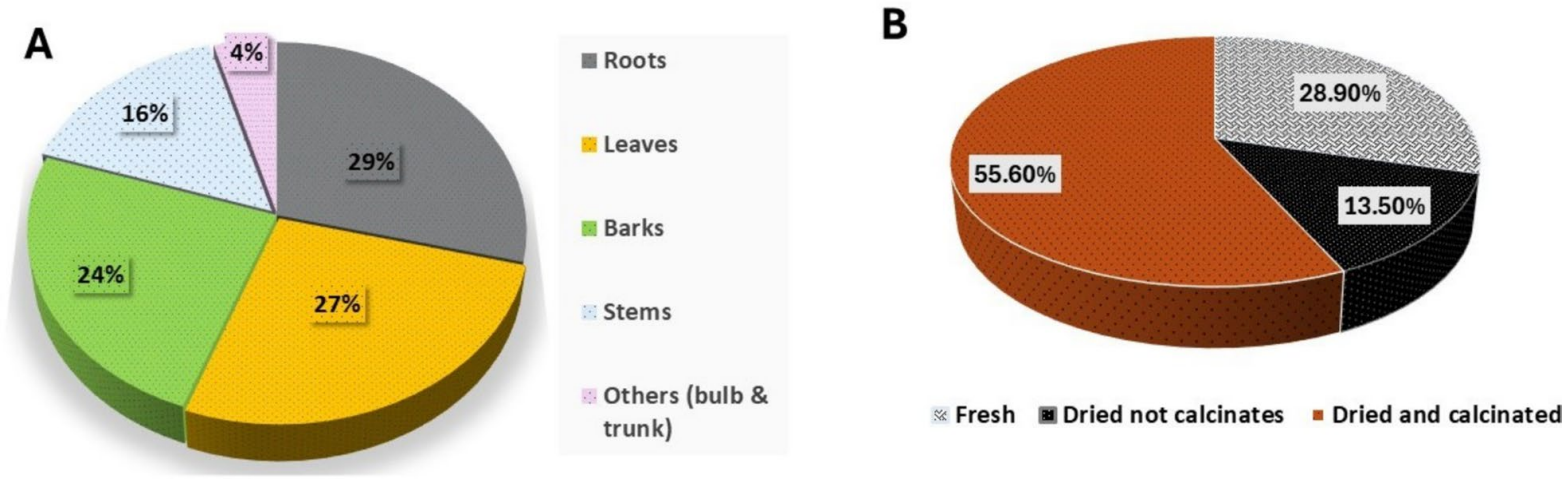
**a** = plant parts used, **b** = initial plant material processing

Roots were the most commonly used plant part in antivenom preparations (29%), followed by leaves (27%), bark (24%), stems (16%), and other parts (4%) (Fig. 3a). The predominant form of medicinal plant preparation in the region involved dried and calcined material (55.6%) (Fig. 3b), whereas fresh plant material was used in only a limited number of cases (13.5%) (Fig. 3b). The dried but uncalcined plant parts accounted for 28.9% of the initial processing of the plant material (Fig. 3b).

### Frequence of citations (FC) and relative frequency of plant citations (RFC)

Table 8 presents the relative frequency with which each plant species was mentioned by traditional health practitioners in relation to the preparation of antivenom recipes.

**Table 8:** Frequency of citation and Relative frequency of citations for each plant species

Plant species	Relative Frequency of Citation (RFC) (%)
*Ageratum conyzoides* L.	3.3
*Allium cepa* L.	3.3
*Annona senegalensis* Pers	10
*Bombax costatum* Pellegr. & Vuillet	3.3
*Bridelia ferruginea* Benth	3.3
*Calotropis procera* (Aiton) Dryand	3.3
*Carissa spinarum* L.	3.3
*Combretum micranthum* G. Don	3.3
*Cymbopogon giganteus* Chiov	6.7
*Diospyros mespiliformis* Hochst. ex. A.DC	3.3
*Eleusine indica* (L.) Gaertn	3.3
*Entada africana* Guill. & Perr	3.3
*Euclea divinorum* Hiern	3.3
*Gardenia erubescens* Stapf & Hutch	3.3
*Gardenia ternifolia* Schumach. & Thonn	6.7
*Guiera senegalensis* J.F.Gmel	6.7
*Gymnosporia senegalensis* (Lam.) Loes	3.3
*Heteroscyphus giganteus* (Steph.) Hürl	3.3
*Lannea velutina* A. Rich	3.3
*Mangifera indica* L.	3.3
*Nauclea latifolia* Sm	10
*Flueggea virosa* (Roxb. ex Willd.) Royle	6.7
*Saba senegalensis (A.DC.)* Pichon	3.3
*Securidaca longepedunculata* Fresen	6.7
*Senegalia macrostachya* (Rchb. ex DC.) Kyal. & Boatwr	3.3
*Sterculia setigera* Delile	3.3
*Vitellaria paradoxa* C.F.Gaertn	10
*Vitex doniana* Sweet	3.3
*Zanthoxylum zanthoxyloides* (Lam.) B.Zepernick & Timler	3.3

The plant species with the highest relative frequency of citation (RFC) were *Annona senegalensis* Pers., *Nauclea latifolia* Sm., and *Vitellaria paradoxa* C.F. Gaertn., all showing a RFC of 10%.

## Discussion

Snakebite envenomation is common in Burkina Faso, as it is in many tropical countries, and constitutes a major medical emergency. In traditional societies, snakebites are not always perceived as accidental encounters between a person and a snake but rather as the result of a curse or malevolent spell cast by an adversary [21]. This belief system may partly explain the widespread reliance on traditional remedies among rural populations. Moreover, the prohibitive cost of healthcare, particularly for communities living in precarious socioeconomic conditions, further encourages the use of traditional medicine [4].

This ethnobotanical study highlights the diversity of traditional health practitioners (THPs) involved in the treatment of snakebite envenomation in Burkina Faso, particularly in the Eastern, Centre-South, and South-Western regions. However, the study is not without limitations. Potential biases may have affected the responses of THPs, as their accounts could have been influenced by memory recall or a tendency to provide socially desirable answers. Furthermore, the scope of quantitative analysis was limited, as the data collected permitted the calculation of only one ethnobotanical index, namely the Relative Frequency of Citation (RFC).

Data analysis revealed the high level of community recognition enjoyed by these practitioners. Indeed, more than 60% of respondents in each region reported knowing at least one THP and stated that they had either personally experienced or witnessed the efficacy of their treatments. These observations are consistent with those of Bamogo et al. (2021), who documented the crucial role of traditional knowledge and phytotherapy in West African healthcare systems [22].

A majority (90%) of the THPs surveyed were male, with over half reporting more than twenty years of experience treating snakebite cases and were illiterate. Similar demographic characteristics have been observed in other studies conducted in Burkina Faso and across the West African subregion. For example, Bamogo et al. (2023) reported that 95% of THPs in the western part of Burkina Faso were male, with 76.1% being illiterate and 70.1% being over the age of 50. Similarly, in Benin, 97.4% of the THPs surveyed were male, with those over 50 years of age constituting 51.3% [21]. This male dominance may be attributed to sociocultural norms that limit female participation in traditional healing, except for gender-specific and paediatric ailments such as malaria, haemorrhoids, infertility, and menstrual disorders [21]. The high proportion of experienced THPs may also reflect the selection criteria, which prioritised those with strong reputations within their communities.

In terms of diagnostic practice, 60% of THPs rely on observing the bite site for signs such as puncture marks, haemorrhage, fang impressions, or specific odours, whereas 26.7% of THPs are based on assessments of clinical signs. Typically, traditional West African medicine does not involve systematic diagnosis combining patient-reported symptoms with laboratory or imaging techniques [23]. Instead, diagnosis is based on symptomatic observation and empirical knowledge. This aligns with the findings of this study, where more than 85% of the THPs used physical and symptomatic criteria. The most commonly reported clinical signs included haematological symptoms (e.g., haemorrhage, haemoptysis, and pallor), followed by inflammatory signs (pain, oedema, necrosis). These manifestations are consistent with viperine syndrome, which encompasses local inflammation, hypotension, and hemorrhagic symptoms. Severe envenomation may even lead to ischaemic or haemorrhagic stroke [24]. These findings are consistent with Roman’s inventory of venomous snakes in Burkina Faso, which reported a predominance of the Viperidae family, notably *Causus maculatus* and *Echis ocellatus*, as the main species responsible for envenomation [25]. In contrast, cobraic syndrome typically begins with paresthesia, fasciculations, muscarinic symptoms, ptosis, and cranial nerve involvement, followed by ascending areflexic paralysis and, ultimately, respiratory failure [26].

One of the key aspects of this study is the relationship between THPs and the formal healthcare system. The majority of practitioners reported having received training or awareness sessions from health facilities but less of the half referred patients to biomedical centres when traditional treatments failed. In some cases, the referral process is reversed. This emerging collaboration is promising and could be strengthened through integrative health strategies aimed at bridging traditional and modern medicine.

With respect to the therapeutic scope of the remedies, 60% were reported to be effective against all snake species, whereas 40% were specific to viperine envenomation. This specificity likely reflects the high prevalence of Viperidae species in the studied regions. As venom composition and clinical manifestations are species dependent [27], such practitioner beliefs may derive from empirical familiarity with local envenomation patterns.

For the preparation methods, remedies were mainly in powdered form, primarily plant based. This contrasts with findings from Côte d’Ivoire, where Tra Bi Boli et al. (2024) reported that pastes prepared from freshly ground leaves or whole plants were more commonly used [28]. Roots were the most frequently used plant parts, followed by leaves and bark. These findings differ from those of Yosef S. et al. (2025), who reported that leaves are the most commonly used parts in Ethiopia for treating human ailments [29]. Roots and leaves are known to retain a wide range of bioactive secondary metabolites long after harvesting, which may account for their frequent use in traditional medicinal preparations [30]. The predominance of root use observed in the present study, although slight, raises environmental concerns, as root harvesting threatens the survival of individual plants [31–33] and, consequently, the preservation of the species in the region. In most studies reported in the literature, leaves are the most commonly used plant parts [34–37], which offers the advantage of allowing regeneration of the plants after collection [38]. Moreover, their study indicated that fresh plant material was predominantly used (67%) [39], whereas in the present study, dried material was predominant. An intriguing aspect of this study was that more than half of remedies were dried and calcined. In fact, most ethnobotanical survey findings reported the predominant use of fresh plant material in traditional medicine [40–43]. This raises questions regarding their mechanism of action. Do they act through residual active compounds, the adsorption of venom, or their properties being altered by calcination? Some authors have investigated the impact of calcination on the bioactivity and phytochemical profile of plant materials. For example, Zhukovets and Özcan (2020) reported reduced antioxidant activity in calcined *Zingiber officinale* Roscoe extracts compared with their raw forms [44]. Conversely, Xue et al. (2022) reported that carbonisation decreased gingerol levels but increased shogaol and gingerone concentrations [45].

Topical application was the predominant route of administration. Powders are often applied directly to incisions at the bite site, a practice also reported by Bamogo et al. (2023) in Burkina Faso’s Hauts-Bassins and Southwest regions [30]. However, this method is discouraged because of the risks of hemorrhage and infection [46]. Furthermore, given the acute nature of snakebite envenomation, the effectiveness of dermal application is questionable because of variability in the dermal absorption of active compounds.

With respect to pharmacological claims, 46.7% of the THPs believed that their remedies both neutralised the venom and alleviated its effects. In clinical toxicology, snakebite management typically involves symptomatic treatment and specific antivenom therapy [46]. Unlike biomedical treatment, which combines multiple drugs (anti-inflammatories, antibiotics, haemostatics, etc.) with antivenom [47], traditional treatments tend to be monotherapeutic.

With respect to the plant species used, 29 species from 18 botanical families were recorded, reflecting considerable phytochemical diversity and potential for antivenom drug development. Numerous reviews have reported the use of plants in snakebite treatment: Giovannini and Howes (2017) listed 208 species in Central America; Dharmadasa et al. (2016) documented 341 species from 32 families in Sri Lanka; Okot et al. (2020) reported 60 species from 28 families in Uganda; and Dossou et al. (2021) identified 109 species from 51 families in Benin [21, 48–50]. In this study, the Rubiaceae family was the most represented, followed by the Euphorbiaceae, Annonaceae, Apocynaceae, Poaceae, and Sapotaceae families. Several of these species are also used in Mali [51], Nigeria [52], and Kenya [53]. *Annona senegalensis* Pers., *Nauclea latifolia* Sm., and *Vitellaria paradoxa* C.F. Gaertn. Were cited in all three studied regions and had the highest relative citation frequencies (10% each). Similarly, Bamogo et al. (2023) reported *Annona senegalensis* Pers. as highly cited (14.5%), although *Securidaca longipedunculata* Fresen. was the most frequently mentioned. Phytochemical screening of *Annona senegalensis* Pers. extracts revealed the presence of tannins, flavonoids, saponins, alkaloids, glycosides, steroids, essential oils, anthocyanins, triterpenes, and coumarins [54]. These compounds may confer various biological activities, such as anti-inflammatory, analgesic, antibiotic, and hemostatic effects, which could contribute to the treatment of snakebite envenomation [55].

## Conclusion

This ethnobotanical study underscores the pivotal role of THPs in the management of ophidian envenomations in Burkina Faso, owing to their accessibility, social legitimacy, and the prohibitive cost of conventional medical treatments. This study revealed the use of 29 plant species by traditional health practitioners for the management of snakebite envenomation across these three regions. The most represented botanical family was Rubiaceae. The plant species *Annona senegalensis* Pers., *Nauclea latifolia* Sm., and *Vitellaria paradoxa* C.F. Gaertn. Were consistently identified in all three regions. The wide range of medicinal plants documented highlights the phytochemical potential that could be harnessed to develop alternative therapeutic approaches. However, the predominance of calcined preparations, topical applications, and empirically based diagnostic practices underscore the need for rigorous scientific validation of the efficacy and safety of the remedies identified in this study for the treatment of snakebite envenomation.

In the context of increasing collaboration between traditional and modern medicine in Burkina Faso, the integration of THPs into a multidisciplinary research framework could contribute to the optimisation of treatment protocols. The standardisation of formulations, alongside comprehensive pharmacological and clinical investigations, would constitute a crucial step toward more effective and safer management of ophidian envenomations in rural settings. A scientific approach and the development of methodologies to study the mechanisms by which these remedies act are challenging.

## Data Availability

No datasets were generated or analysed during the current study.
